# Description of a Well Preserved Fetus of the European Eocene Equoid *Eurohippus messelensis*

**DOI:** 10.1371/journal.pone.0137985

**Published:** 2015-10-07

**Authors:** Jens Lorenz Franzen, Christine Aurich, Jörg Habersetzer

**Affiliations:** 1 Department Messelforschung, Senckenberg Forschungsinstitut Frankfurt, Frankfurt am Main, Germany; 2 Department Geowissenschaften, Naturhistorisches Museum Basel, Basel, Switzerland; 3 Department Universitätsklinik für Kleintiere und Pferde, Veterinärmedizinische Universität Wien, Wien, Austria; Raymond M. Alf Museum of Paleontology, UNITED STATES

## Abstract

The early Middle Eocene locality of Grube Messel, near Darmstadt (Germany), is famous for its complete vertebrate skeletons. The degree of preservation of soft tissues, such as body silhouettes, internal organs and gut contents, is frequently remarkable. The present specimen was analyzed for remnants of the reproductive system. Classic anatomy and osteology and high-resolution micro-x-ray were applied to describe the fetus of the European Eocene equoid *Eurohippus messelensis*. Scanning electronic microscopy (SEM) was used for determination of soft tissue remnants. The fetus is the earliest and best-preserved fossil specimen of its kind. The postcranial fetal skeleton is almost complete and largely articulated, allowing the conclusion that the pregnant mare was in late gestation. The apparent intrauterine position of the fetus is normal for the phase of pregnancy. Death of mare and fetus were probably not related to problems associated with parturition. Soft tissue interpreted as the uteroplacenta and a broad uterine ligament are preserved due to bacterial activity and allow considerations on the evolutionary development of the structures.

## Introduction

**Fig 1:**
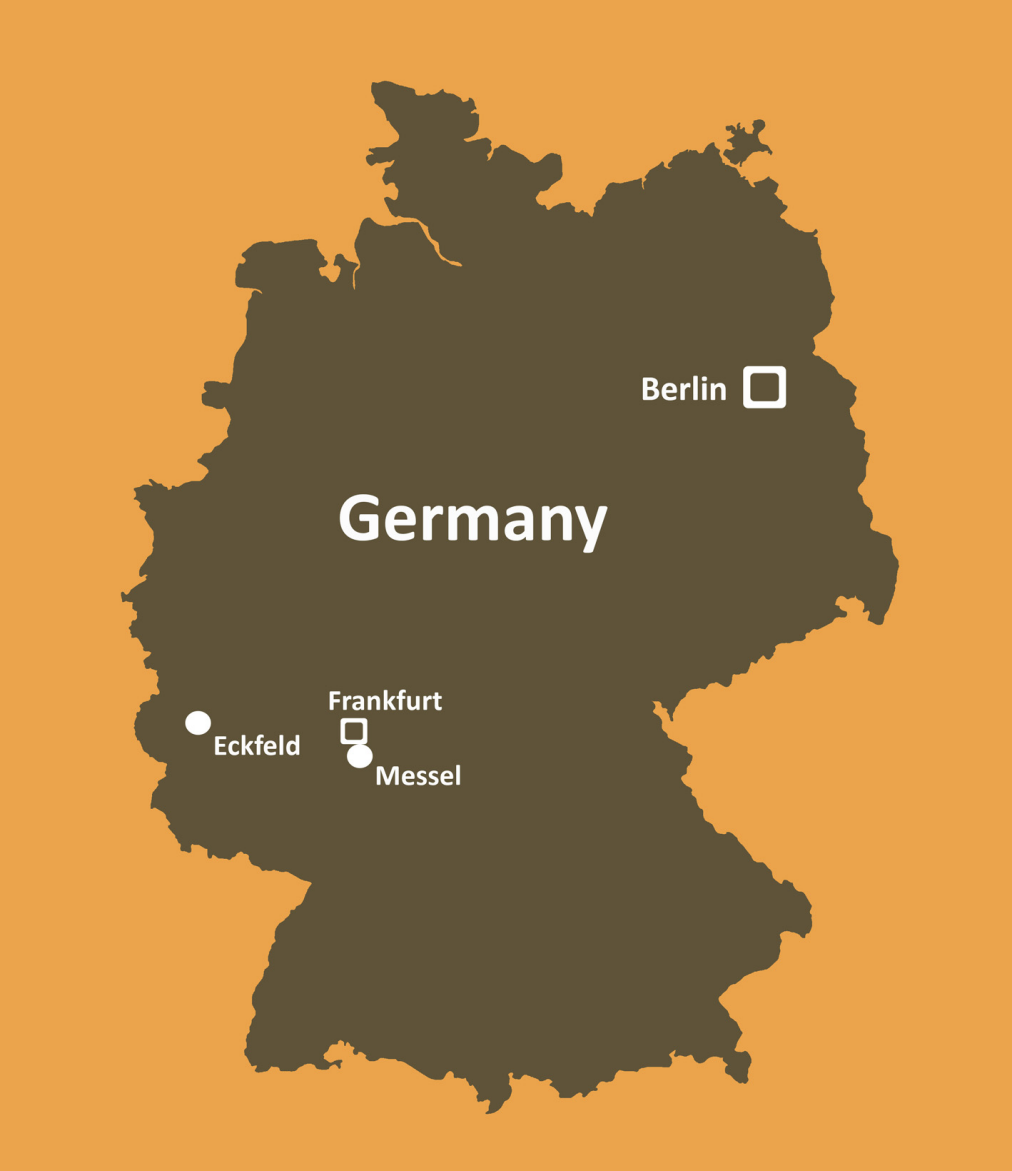
Map of Germany showing the geographic location of Grube Messel and Eckfeld. –Drawing: Senckenberg Forschungsinstitut Frankfurt, Mascha Siemund.

The fossil record of mammals is often restricted to isolated teeth and bones. Less than 2% of localities with fossil mammals have yielded anything more than fragments of jaw material and other bones [1, 2]. Skeletons of pregnant females with fetuses are extremely rare. In Germany, two localities of Eocene age have delivered skeletons of pregnant mares of primitive horses: the maars of Eckfeld in the SW of the Eifel Mountains and of Messel, near Frankfurt am Main and Darmstadt (Fig 1).

Wighart von Koenigswald was the first to describe the skeleton of a mare with fetus from the Grube Messel [3], which was found in 1986. Koenigswald determined the species as *Propalaeotherium parvulum*, which was subsequently synonymized with *Eurohippus messelensis* [4–5]. All pregnant mares found in the Messel quarry represent this taxon, as revealed by the examination of 39 skeletons and their comparison with the lectotype [4].

**Fig 2:**
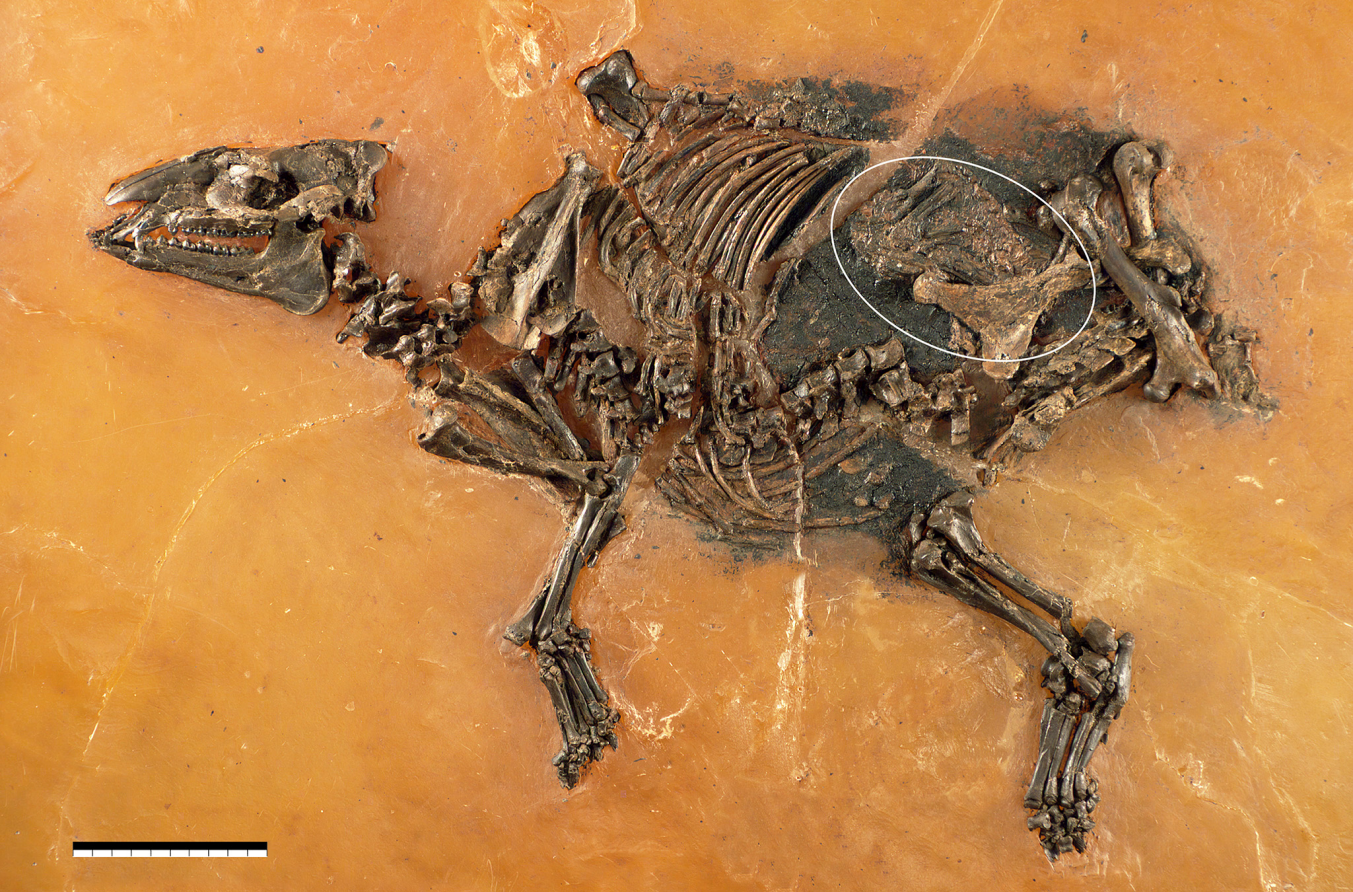
Skeleton of a mare of *Eurohippus messelensis* with fetus (white ellipse). The specimen was discovered and excavated by a team of the Senckenberg Research Institute Frankfurt at the Grube Messel (Germany; inv. no. SMF-ME-11034), shoulder height ca. 30 cm, scale = 10 cm.–Photo: Senckenberg Forschungsinstitut Frankfurt, Sven Tränkner.

Maier [6] described four mares with fetuses from the Grube Messel, whereas Franzen [4] presented eight pregnant mares from Messel in a monograph on the Eocene Equoidea. Six of them contain fetal skeletons, of which four are more or less disarticulated and scattered. The Darmstadt specimen (HLMD-Me 8989) is relatively well preserved, although the thorax is rather disarticulated and the lumbar vertebrae are missing. By far best preserved is the fetus of a mare that was discovered and excavated by a team of the Senckenberg Research Institute Frankfurt in 2000 (Fig 2). It is archived in the Institute’s Department of Messel Research under the inventory no. SMF-ME-11034 and has been briefly mentioned by several authors [4, 7–10]. We now describe it in detail for the first time and evaluate its paleobiologic and evolutionary significance. The skeleton is virtually complete and mostly articulated. The soft tissue is outstandingly well preserved.

All permits necessary for the study were obtained and the work fully complies with all relevant regulations.

## Description

The use of anatomical terms in relation to the skeleton of the mare is sometimes misleading, because when the skeleton was embedded on the bottom of the Eocene Lake Messel parts of it came to lie on one or the other side of the vertebral column, irrespective of the side to which they anatomically belong. The right zeugo- and autopodia were deposited to the left of the vertebral column, which was itself situated not dorsally but in the middle of the body (Fig 2). Anatomical terms are thus applied as if the vertebral column were dorsal. With regard to the fetus, terms are used with respect to the fetal and not the maternal skeleton, unless otherwise indicated.

**Fig 3:**
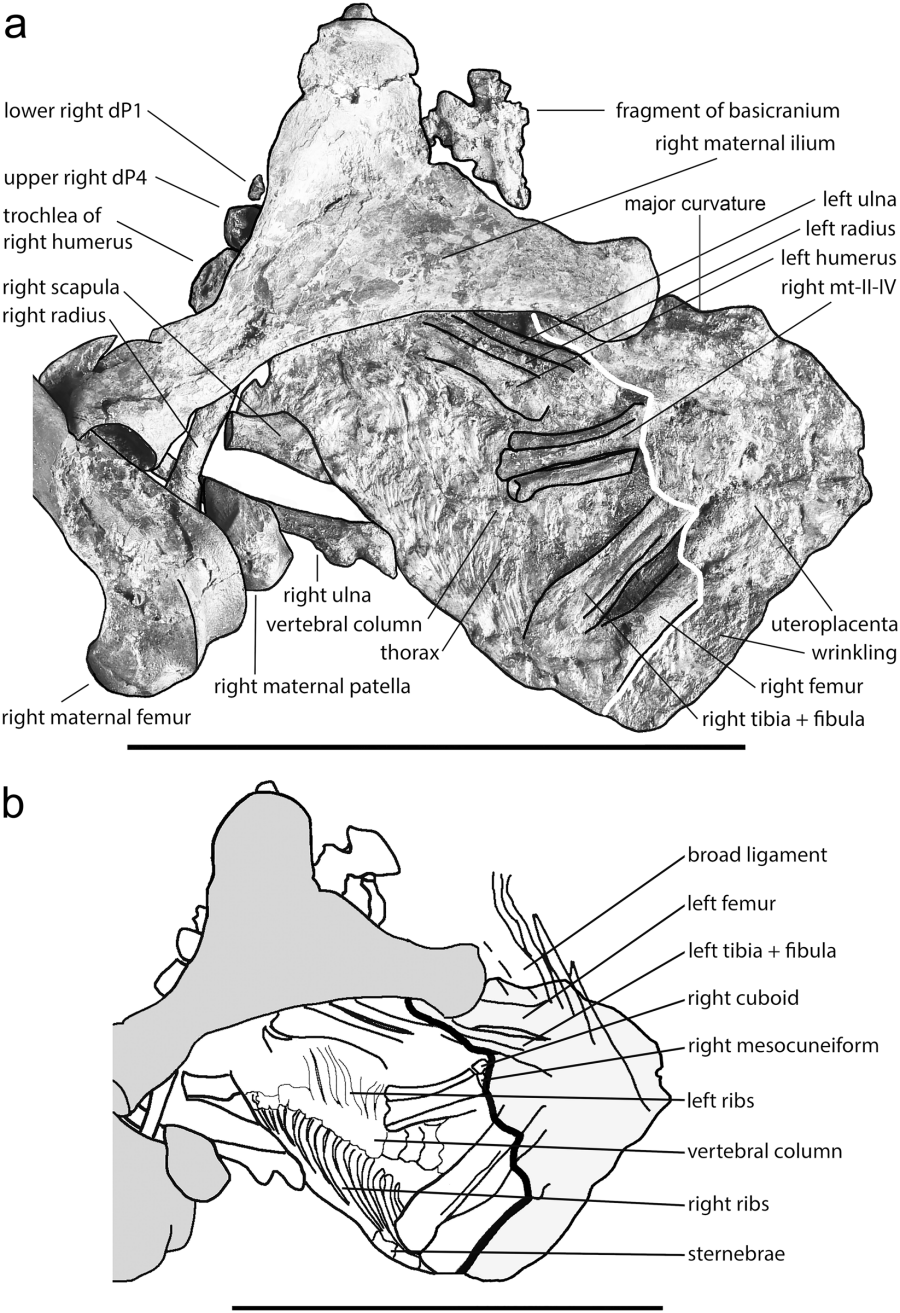
**a) Line drawing of the exposed side of the fetus of *Eurohippus messelensis* based on a reduced-contrast photo as background**. Bones of the mare (black) are shown for orientation. Notice the presence of the uteroplacenta as indicated by a fine wrinkling lateral of the right femur, covering a large part of the fetus. The white line distinguishes the uteroplacenta on the right side from the exposed bones on the left. dP_1_ = first lower deciduous premolar of the right side, dP^4^ = last upper deciduous premolar of the right side.—Photo: Senckenberg Forschungsinstitut Frankfurt, Sven Tränkner; line drawing: Jens Lorenz Franzen. **b) Line drawing of the skeleton of the fetus and adjacent bones of the mare based on a micro-x-ray** (Fig 4). The outline of the uteroplacenta is taken from Fig 3a. Dark grey are the bones of the mares, white those of the fetus. Light grey is the uteroplacenta. Lettering is in Fig 3a except for a few bones and the broad ligament, which are only identifiable on the micro-x-ray (Fig 3b). Scale of a and b = 10 cm.–Micro-x-ray: Senckenberg Forschungsinstitut Frankfurt, Jörg Habersetzer; line drawing: Jens Lorenz Franzen.

**Fig 4:**
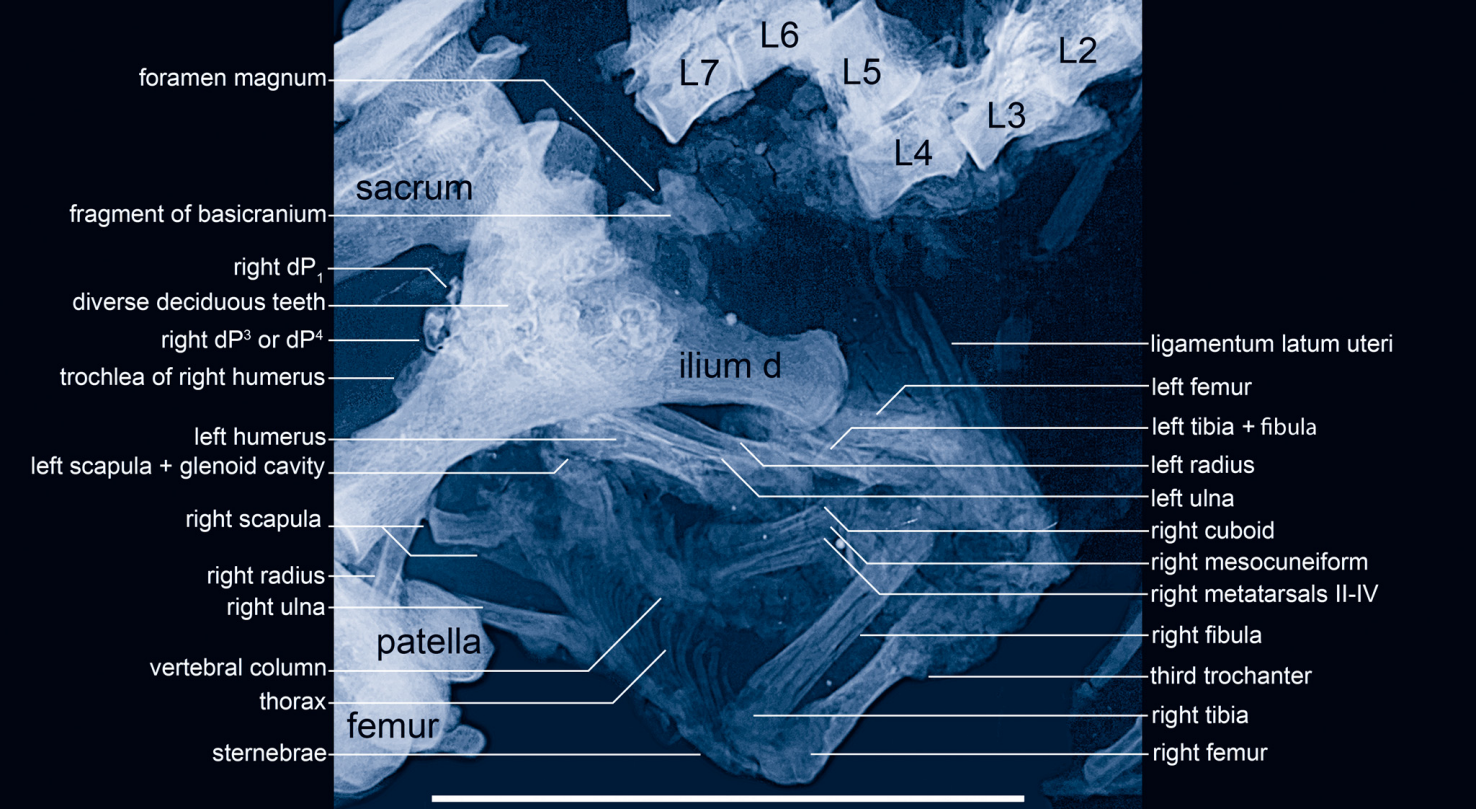
The fetus analyzed in detail by high-resolution micro-x-ray. Bones of the mare are indicated by black lettering, bones and teeth of the fetus by white lettering. L2-7 = lumbar vertebrae 2–7 of the mare. Scale = 10 cm.–Anatomical interpretation: Jens Lorenz Franzen; micro-x-ray: Senckenberg Forschungsinstitut Frankfurt, Jörg Habersetzer.

The fetus is preserved as a compact ellipsoid accumulation of bones located cranioventrally to the maternal pelvis (Figs 2–4). From the surface, it is almost impossible to distinguish individual bones with certainty (Fig 3a). The fetal bones are greatly compressed and the joints were not fully ossified. It seems likely that the pelvic area is still covered by remnants of the uteroplacenta. The identification of individual bones thus stems primarily from the micro-x-ray (Fig 4) and is based on size and proportions, special features—such as the third trochanter on the right femur—and on the fact that most of the bones are still articulated at nearly the original positions.

Anatomically, the thoracic part of the fetal vertebral column is situated on the ventral side of the mare near the ventral uterine wall (Figs 3 and 4). The thorax is seen from its ventral side. The vertebral column appears within and in articular contact with the ribs of the left and right sides. The lumbar vertebrae 1–3 occur distal to the right tibia and vertebra no. 3 vanishes below the right tibia. Cranially, the vertebral column points in a caudal direction with respect to the mare. The right ribs end distally at segmented bones that obviously represent the sternebrae, whereas those of the left side submerge below the left forelimb of the fetus. Even on the micro-x-ray, no details of the fetal pelvic area are discernible. The hind limbs are stretched apart, the left one to the dorsal side and the right one to the ventral side of the uterus. The right femur displays a third trochanter near its proximal end, which is situated cranioventrally with respect to the mare. Elongated bones, a massive and a thin one, occur almost parallel to the femur and to each other. They are interpreted as the right tibia and fibula. The right metatarsals II-IV originate near the distal end of the right tibia at an angle of 35°. Their proximal ends are better ossified than the distal ones. Proximal of metatarsal IV occurs the cuboid and of metatarsal III the mesocuneiform, whereas the calcaneus and talus submerge below a flat structure, which is suggested as remnant of the uteroplacenta (actually p. 9). Phalanges are not discernible. The left femur and the left lower leg appear to lie parallel to each other on the dorsal side of the uterus. They are only recognizable on the microradiograph (Figs 3b and 4). We cannot identify the calcaneus, talus or other tarsals, metatarsals or phalanges of the left side.

The left humerus of the fetus is encapsulated between the right ilium of the mare proximally and the right metatarsals of the fetus distally. It is identifiable on the micro-x-ray because its caput articulates proximomedially with the glenoid cavity of the left scapula, which is situated between the ribs of the left and the internal uterine wall on the right side, so that most of it is hidden. Distally, the left humerus is not completely ossified. The same is true of the proximal parts of the left radius and ulna, which are situated laterally and parallel to the left humerus. Distally, these bones disappear below the right ilium of the mare.

In contrast to the left side, the bones of the right shoulder and forelimb are disarticulated and dislocated. The proximal part of the right scapula is exposed from its medial aspect (Fig 3a). The spina scapulae and its tuberosity appear only on the microradiograph (Fig 4) because the structures are situated on the lateral side. Most of the medial side of the right ulna is visible on the ventral side of the uterus, its olecranon pointing cranially with respect to the mare. Knob-like structures proximal and distal from what is considered to be the trochlear notch seem to correspond to the anconeal and coronoid processes, respectively (Figs 3 and 4). The distal end of the ulna vanishes below the right patella of the mare. The diaphysis of the right radius appears between the right patella, femur and ilium of the mare, whereas the right humerus of the fetus seems to be represented only by its trochlea, which is partly visible medially of the medial edge of the right maternal ilium (Fig 3a). Only on the micro x-ray (Fig 4) are its typical steep medial and lateral contours and the smooth convex articulation for the capitulum of the radius visible in craniocaudal view.

The cranium of the fetus is obviously crushed because its fragments are scattered. Part of the basicranium including the foramen magnum appears craniodorsally to the iliac crest of the mare (Figs 3 and 4). The foramen magnum points in dorsocaudal direction (with respect to the mare). Other bones of the skull are not recognizable and are presumably hidden below the right ilium of the mare.

**Fig 5:**
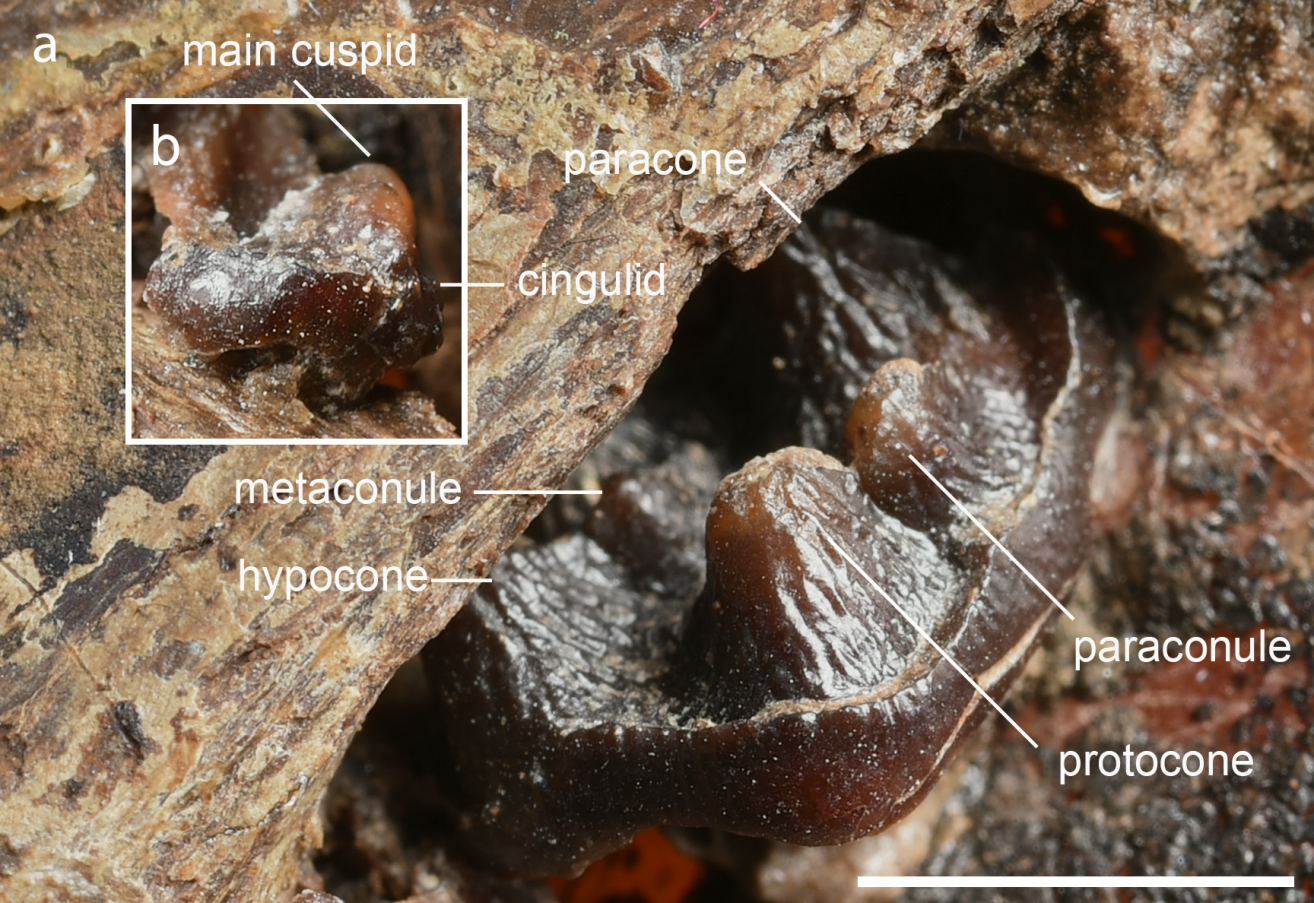
a) Occlusal surface of the last upper deciduous premolar (dP^4^) of the right side seen from its mesiolingual corner. b) Buccal aspect of the right dP_1_. Scale of a and b = 5 mm.—Photo: Senckenberg Forschungsinstitut Frankfurt, Sven Tränkner.

Most of the deciduous teeth are visible only on the micro-x-ray because they are covered by the right ilium of the mare. Even here their contours are vague, so that details cannot be determined (Fig 4). A right upper deciduous premolar appears caudally behind the maternal ilium (Fig 5a). It displays an occlusal pattern consisting of parastyle, paracone, paraconule, protocone, metaconule and hypocone. The metacone is covered by the right ilium of the mare and not visible. The size and arrangement of these cusps indicate a right dP^4^. Contrasting with dP^3^, the mesiolingual corner of dP^4^ does not retreat and the parastyle does not protrude in mesial direction. The fragment of another deciduous tooth lies craniodorsally to the dP^4^ and outside the maternal ilium (Fig 5b); we interpret it as right dP_1_. It displays a convex buccal wall, which shows one main cuspid. Distal to this is a postcristid that ends at the longitudinally short talonid, followed distally by a tiny tip of the cingulid. The buccal wall is near its base surrounded by a vestigial ectocingulid, which ends mesially in a tiny tuberculid. The tooth is biradicular. Seen from the buccal side, both roots are diverging and stick still in their alveoles.

## Measurements

The state of preservation of long bones does not allow exact measurements, so the values of the fetus represent estimates compared to corresponding measurements of the mare [4].

**Table 1:** 

measurements (mm)	fetus	mare
maximum length of right femur	45	137.8
maximum length of left humerus	43	103.1
maximum length of right tibia	49	120.5
maximum length of right metatarsal IV	20	46.2
maximum length of right dP_1_	3.5	4.7

## Preservation of the Reproductive Tract

**Fig 6:**
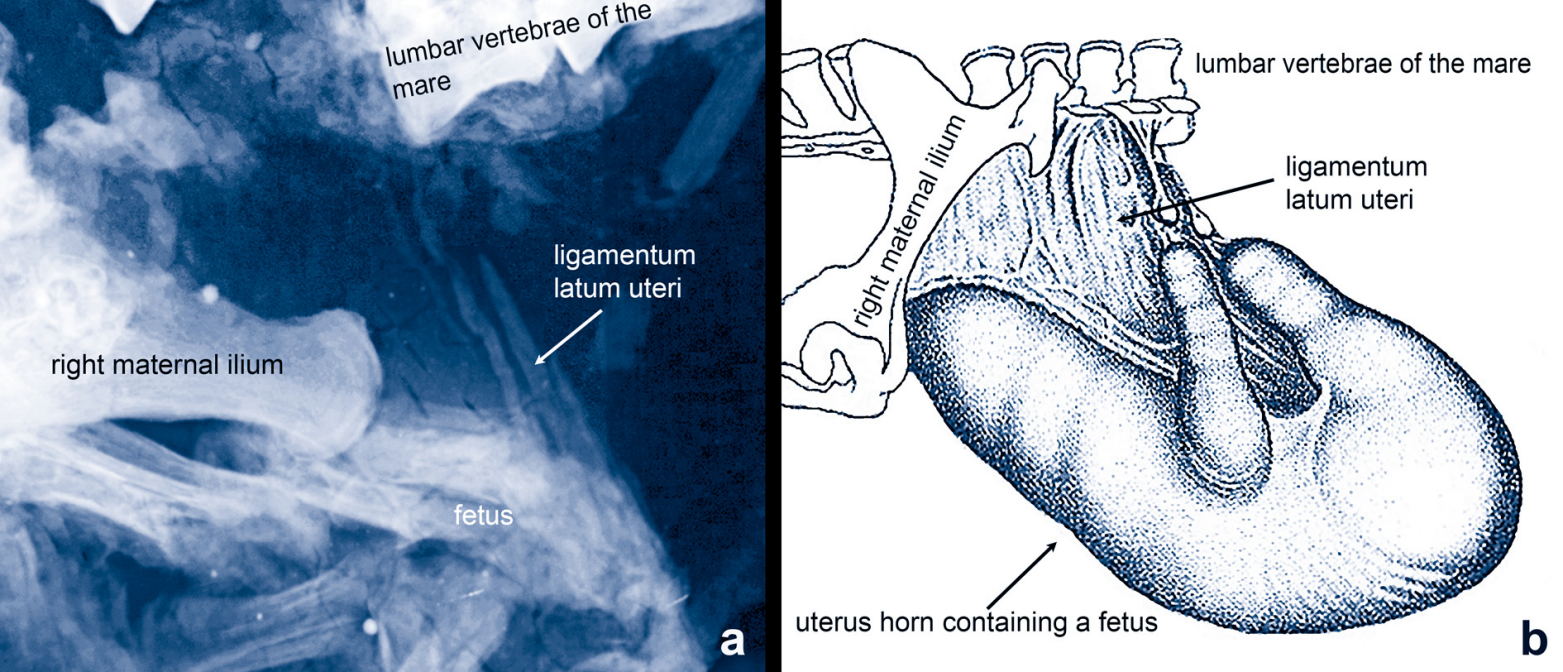
Identification of the broad ligament (ligamentum latum uteri). a) The broad ligament in the fossil mare from the Grube Messel. Sacrum and lumbar vertebrae (L6-7) belong to the mare. Not to scale.—Micro-x-ray: Senckenberg Forschungsinstitut Frankfurt, Jörg Habersetzer.–b) Position and morphology of the broad ligament (ligamentum latum uteri) attaching the uterine horn containing the fetus to the lumbar vertebrae and the pelvis of a modern horse (from Benesch 1957).

The micro-x-ray shows a conspicuous grey shadow between the fetus and the lumbar vertebrae of the mare (Fig 6a). The shadow is partially dispersed into stripes, which run parallel to one another and frame longitudinally elongated holes. Their structure points dorsocaudally to maternal lumbar vertebrae 4–7. In its morphology and position the structure corresponds closely to the broad ligament (ligamentum latum uteri) of recent mares of *Equus caballus* (Fig 6b), where it attaches the uterine horn to the lumbar vertebrae and the pelvis [11]. We can eliminate the possibility that the structure is an artefact of the preparation [12]. An alternative interpretation as abdominal muscle can be excluded because of different position and direction. The musculus transversus abdominis extends in vertical direction from the lumbar vertebrae and the pelvis down to the ventral midline of the abdomen, whereas the musculus obliquus abdominis stretches from the tuber coxae to the last rib and rib cartilage.

**Fig 7:**
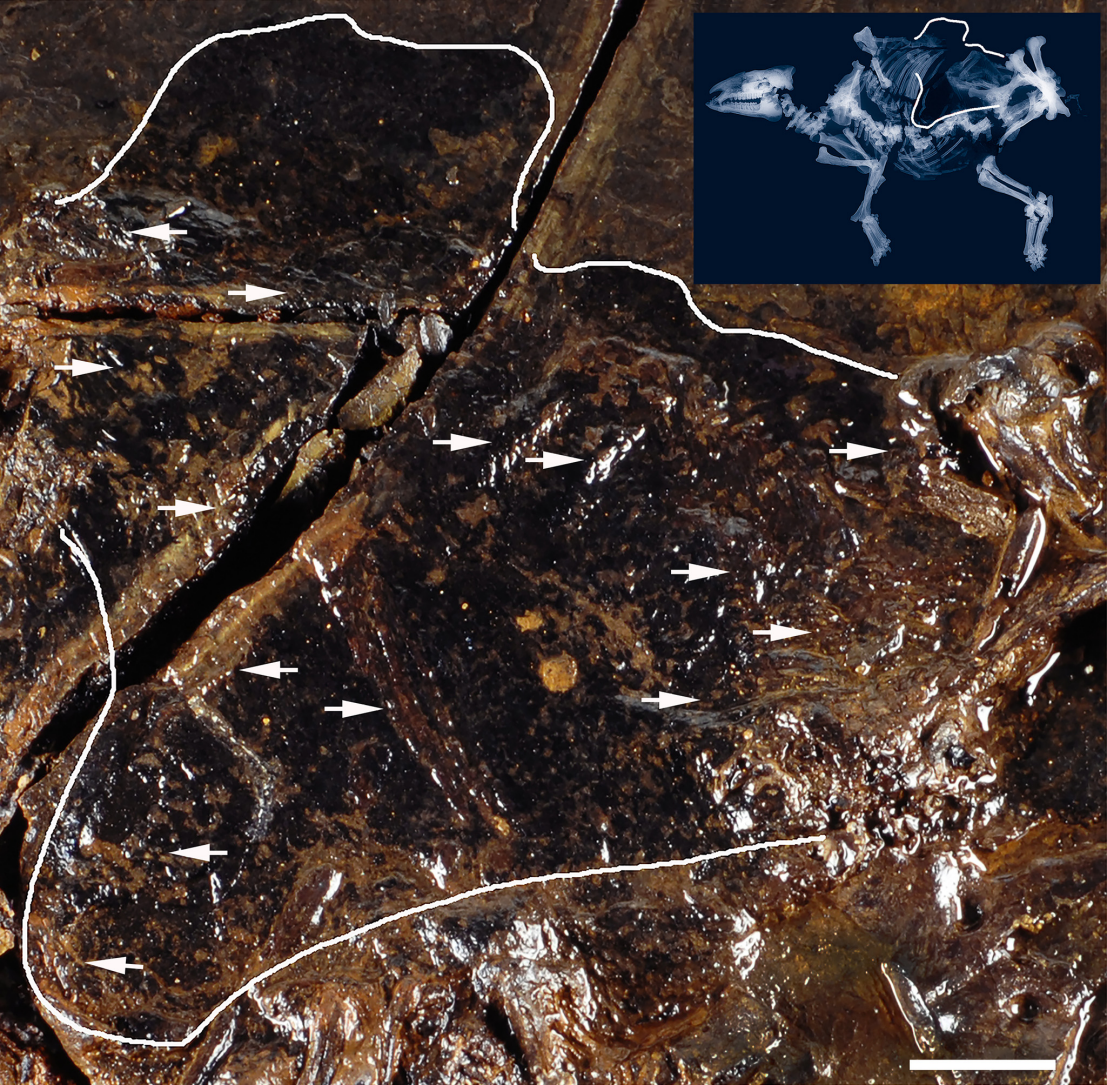
Identification of the uteroplacenta. The photograph of the original upper side of the fossil horse was taken before transfer into epoxy resin during preparation. It displays a black shadow (edged in white) covering the fetus. Arrows point to wrinkling structures, which appeared on the external wall of the uterine horn after accidental rupture. Scale = 5 cm. Right upper corner: for orientation, the outline of the uteroplacenta is shown on a micro-x-ray of the whole skeleton of the mare.—Photo: Senckenberg Forschungsinstitut Frankfurt, Sven Tränkner; micro-x-ray: Senckenberg Forschungsinstitut Frankfurt, Jörg Habersetzer.

As indicated by the compact ellipsoid accumulation of articulated fetal bones that are nearly in the original position, the fetus was still enwrapped in the uteroplacenta (pregnant uterus, lined by the allantochorion) when the carcass fell to the bottom of the Eocene Lake Messel. Photographs of the original upper side of the fossil before transfer into epoxy resin (during preparation; see [12]) show that the fetus was covered by a black shadow (Fig 7). At Messel, such black shadows are typical of preserved soft tissue [13–14], although we are not dealing with direct preservation but with a by-product of bacterial metabolism. In the Eocene Lake Messel, bacterial decomposition of soft tissue produced carbon dioxide, which reacted with dissolved iron from the lake water to precipitate iron carbonate minerals. The bacteria became coated with a thin layer of Fe-carbonate (siderite) and autolithified. A thin and porous bacterial mat developed, which exactly followed the lines of contact between the decomposing soft tissue and the sediment. The mat was subsequently infiltrated by organic material (e.g. kerogene), staining it black [14].

**Fig 8:**
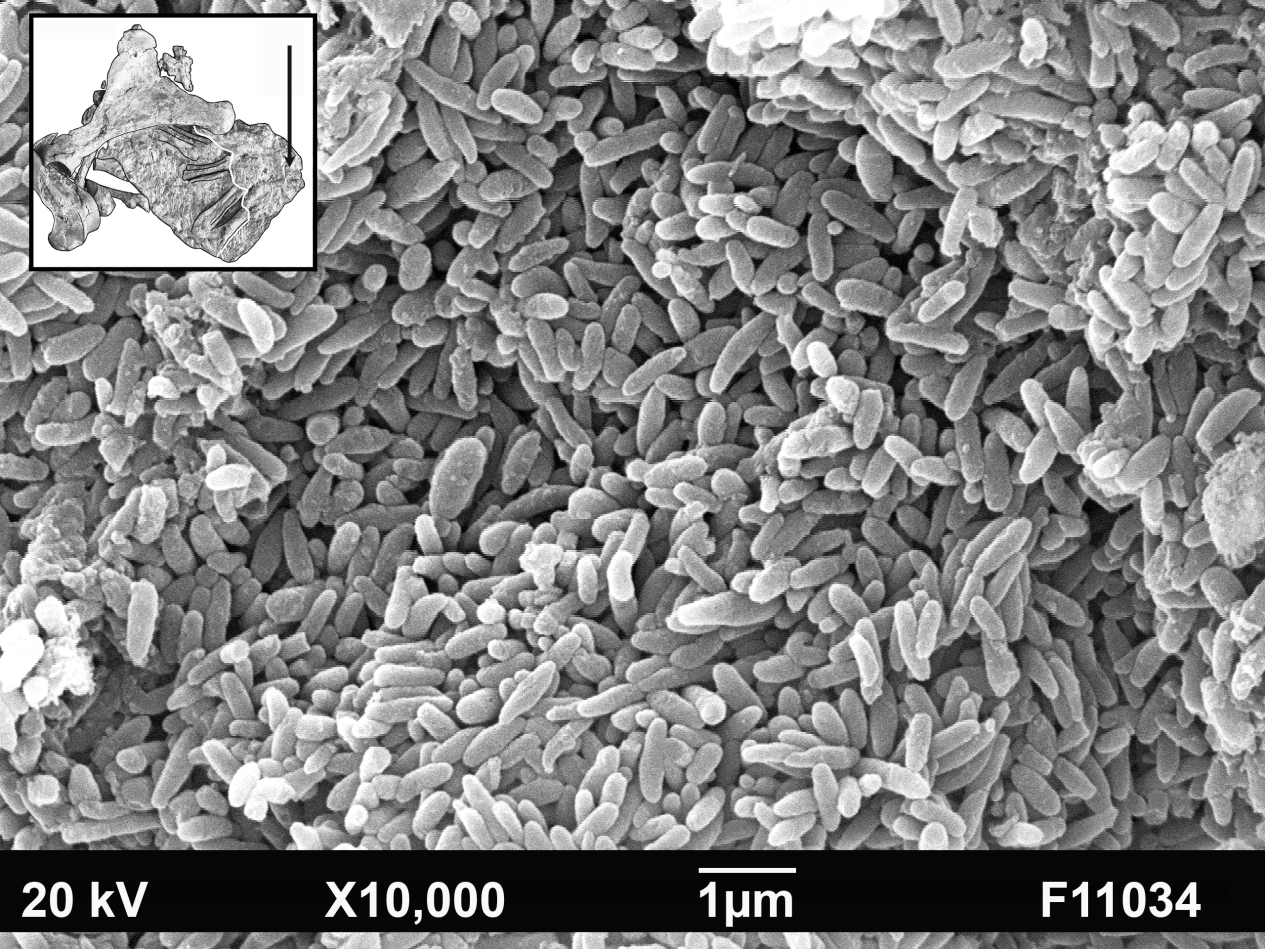
Photograph with Scanning Electronic Microscope (SEM) of the yellow structure on the exposed side of the fetus. Notice the tiny rod-shaped bodies, which correspond in size and morphology with bacteria. Insert: arrow points to the sampling site. Scale = 1 μ.–SEM: Senckenberg Forschungsinstitut Frankfurt, Renate Rabenstein.

**Fig 9:**
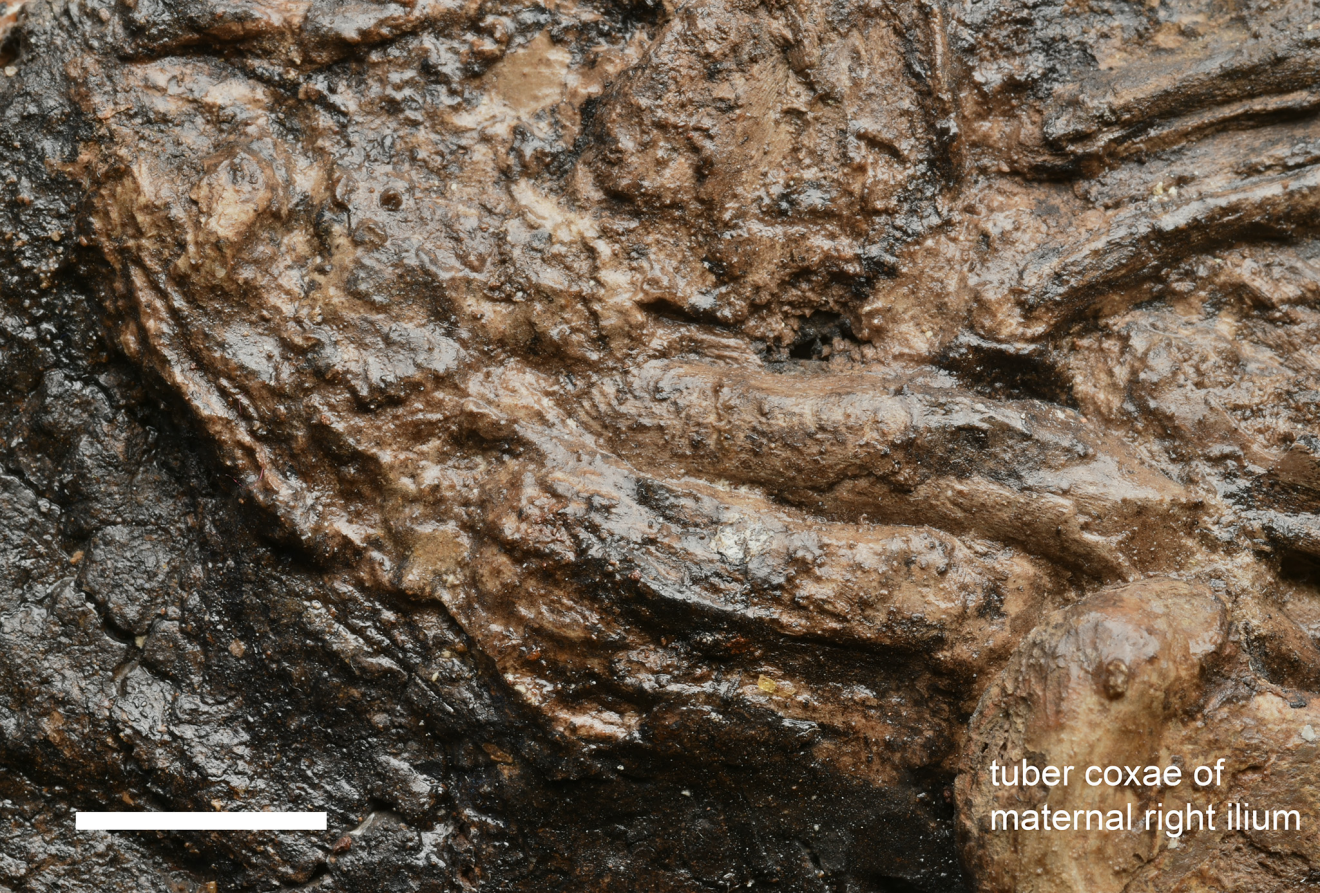
Close-up of the dorsal edge of the uteroplacenta displaying a characteristic folding known as “major curvature” in extant horses. For orientation see Fig 2. Scale = 5 cm.—Photo: Senckenberg Forschungsinstitut Frankfurt, Sven Tränkner.

We assume that the black shadow in the pregnant mare corresponds to the uteroplacenta of the pregnant uterine horn because of its size and position. The extent of the uteroplacenta exceeds considerably the accumulation of articulated fetal bones (Fig 7). This corresponds with the situation in extant pregnant mares. A fine wrinkling that covers almost the entire surface (Fig 7) supports our interpretation. In extant periparturient horses, a wrinkling of the uteroplacenta occurs physiologically when the uterine cavity has been emptied at parturition. It is a result of myometrial contractions and characterizes the start of uterine involution after foaling [15–16]. A wrinkling of the uteroplacenta is also observed in the infrequent case of an accidental rupture of the uterine wall during late pregnancy. We conclude that a traumatic rupture of the uterine wall of the Messel mare at or shortly before death and its subsequent fossilization may have resulted in contraction of the uterine wall but not in expulsion of the fetus from the chorioallantois. Interestingly, a similar wrinkling appears on the exposed side of the fetus lateral to the right fetal femur, and a yellow layer extends over the whole pelvic area and even the bones of the left hind limb. Only some bones, such as the thorax, the right hind limb and the left forelimb are directly visible (Fig 3a). When we applied SEM to analyze the structure, tiny rod-shaped bodies corresponding in size and shape with bacteria were identified (Fig 8). We suggest that the structure represents a part of the uteroplacenta on the exposed side, where it is obviously preserved by bacterial metabolism. A structure, similar to the major curvature of the uterus in recent horses corroborates this interpretation (Fig 9). The fact that most of the bones of the postcranial skeleton of the fetus are still articulated in the original position indicates that even the amnion, which is the part of the placenta directly enwrapping the fetus, was preserved when the dead mare lay down on the bottom of the lake.

Franzen has described a comparable case of a fossilized pregnant mare of the equoid *Propalaeotherium voigti* from the Middle Eocene Maar of Eckfeld, which showed “a hard undulated bright-grey crust enwrapping the bones of a fetus” [17]. This was the first case of an uteroplacenta preserved with the fossilized skeleton of a placental mammal. We now describe the second case, which is much better preserved than the first one despite being about 2 million years older.

## Determination of the Ontogenetic Stage of Development

**Fig 10:**
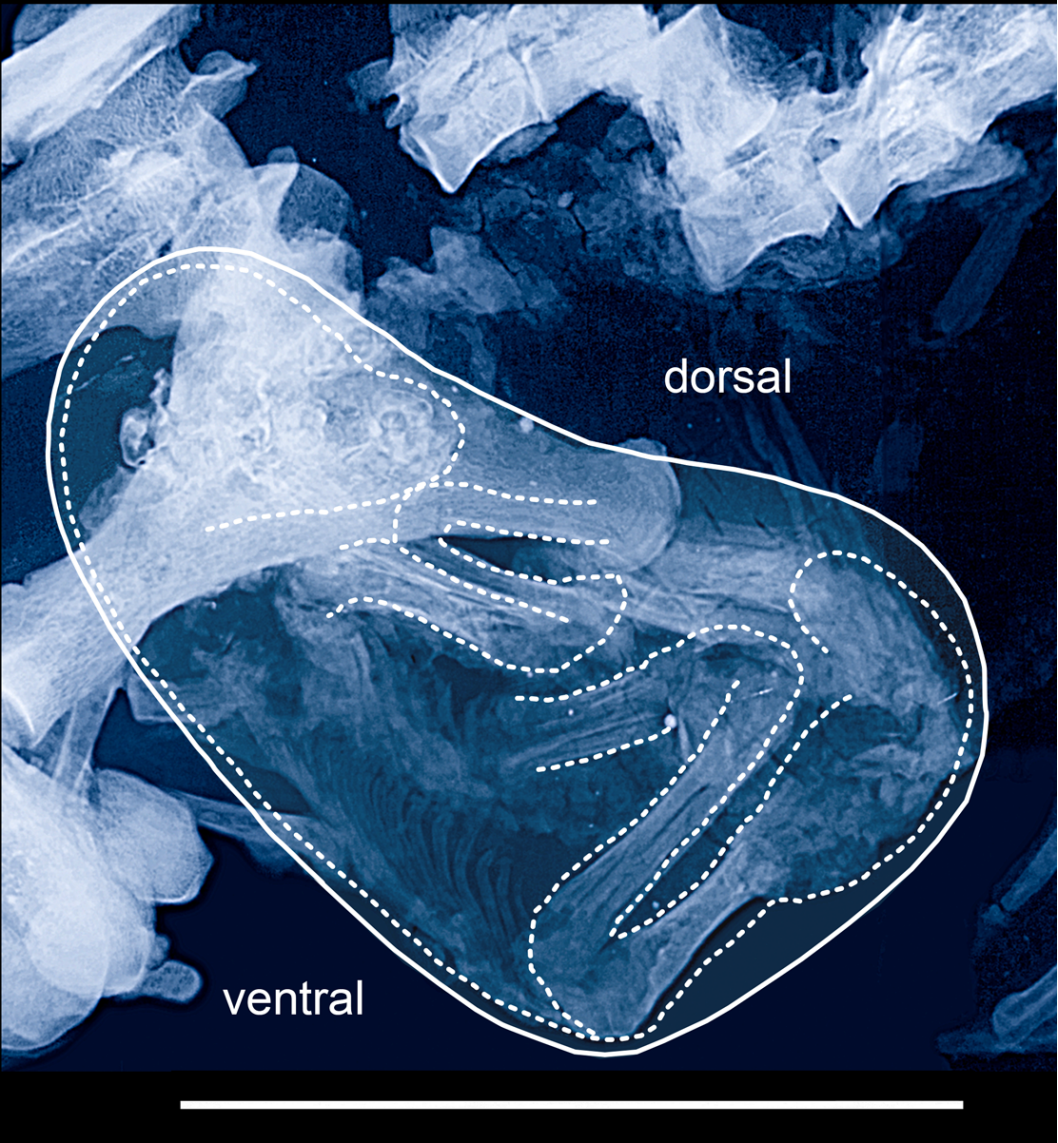
Reconstruction of the original position of the fetus. The reconstruction refers to the almost completely preserved and articulated fetal skeleton as indicated by high-resolution micro-x-ray. Scale = 10 cm.–Reconstruction: Jens Lorenz Franzen; drawing: Mascha Siemund.

Based on the almost completely preserved and articulated skeleton of the fetus, it is possible to reconstruct the fetus’s original position (Fig 10). Its back is situated ventrally and the limbs point dorsally with respect to the vertebral column of the mare, whereas the head is close to the birth channel. By analogy with extant pregnant mares of *Equus caballus* [18], the stage of ossification of long bones and the development of the deciduous dentition (dP^4^ already erupted) indicate that birth was imminent. The forelimbs of the fetus are not yet stretched towards the birth channel and the thorax is not yet turned into a dorsal position. These features are normal until the initiation of parturition [11, 19]. It thus seems that the death of the mare and the fetus were probably not related to problems associated with the birth process.

It is difficult to estimate the approximate gestation length that would have been characteristic for *Eurohippus messelensis*, although it was probably shorter than that of *Equus caballus*, which has a mean gestation length of 11 months. For living mammals, the length of gestation is influenced by body weight: the bigger the body, the longer the gestation period [20]. However, although recent horses and dairy cows have similar body weights (around 600kg), the mean pregnancy length in horses is approximately two months longer than in cows [21]. The body mass of *Eurohippus messelensis* has been estimated as 5–6.5 kg [22]. This is comparable to that of extant blue duikers (*Philantomba monticola*), which have a body weight of 4–6 kg [20] and are of comparable size and similar body structure. Blue duikers live diurnally in the rain forests of central to southern Africa and reproduce only once a year, with gestation ranging from 201 to 213 days [23–24]. We assume that gestation in *Eurohippus messelensis* lasted for at least 200 days. As recent equids, female blue duikers physiologically give birth to only one offspring. The twinning rate at term in modern Thoroughbred racehorses is between 1 and 2% but it is even rarer in ponies and so-called primitive horse breeds [25]. As all pregnant Messel mares found to date were pregnant with a single fetus, we assume that singleton pregnancy was characteristic of *Eurohippus messelensis*.

## Paleobiology

Studies of the skeletons of pregnant mares from Messel confirm that mares had as wide a pelvic channel as recent horses, whereas the pelvic channel of stallions was rather narrow [4, 7–8, 26]. Based on this distinction, 15 of 39 skeletons of *Eurohippus messelensis* from Messel were classified as females and 12 as males, with the sex of 12 not identifiable with certainty. Remarkably, eight of the 15 mares were pregnant, based on the presence of fetal bones. We assume that only the later stages of pregnancy allow conservation of fetal components due to calcification of their skeleton. Earlier stages of pregnancy are probably no longer detectable but may have been present. In recent horses, seasonal reproductive activity results in synchronization of birth of offspring to spring and early summer [21]. In a group of pregnant mares of *Equus caballus*, the developmental stage of the conceptuses does not vary considerably. The finding of similar stages of development of the fetuses in the pregnant *Eurohippus messelensis* mares suggests that similar mechanisms were already present in this species. Comparable with wild living horses [27], most of the adult mares from Messel must have been pregnant. The Munich specimen of *Eurohippus messelensis* (BSP.1985.L62) proves that females could become pregnant before reaching full adulthood: the deciduous dentition of the mare was still functioning although the animal was bearing a fetus [4]. This is also true for recent horses [28].

The specimen of *Eurohippus messelensis* we describe here is not only the oldest but also the best preserved fetus of a primitive equoid. It represents the earliest fossil record of the uterus of a placental mammal and corresponds perfectly with that of living horses. The transport of nutrients through the semiplacenta epitheliochorialis of the horse is less efficient than in humans, rodents, dogs and cats [28]. Nevertheless, epitheliochorial placentation is considered a derived condition and not—as thought before—a primitive one [29]. This is remarkable against the background of the evolutionary changes, particularly of the locomotory apparatus and the dentition, undergone by horses since the Eocene [8]. Evidently, the uterine system developed much earlier, at the latest during the Paleocene but more probably already during the Mesozoic.

## Conclusions

We describe and analyze a fetus of the European Eocene equoid *Eurohippus messelensis* that is significant for the paleobiology of fossil mammals because of the high quality of its preservation. The postcranial skeleton is virtually complete and largely articulated. This makes it possible to reconstruct the position of the fetus, which was normal and corresponds to late gestation of modern horses. It indicates no problem that could have caused the death of the mare and its foal. Soft tissue, such as the uteroplacenta and one broad uterine ligament represent the earliest fossil record of the uterine system of a placental mammal. As usual at Messel, the soft tissue is preserved due to bacterial activity. The excellent preservation proves that the uterine system of mammals developed at the latest during the Paleocene more probably already during the Mesozoic.
